# Model of superior semicircular canal dehiscence: asymmetrical vestibular dysfunction induces reversible balance impairment

**DOI:** 10.3389/fneur.2024.1476004

**Published:** 2024-10-28

**Authors:** Sean S. Hong, P. Ashley Wackym, Damian J. Murphy, Eran Peci, Matthew Y. Kiel, Aaron Tucker, Nicolas L. Carayannopoulos, Shrivaishnavi C. Chandrasekar, Nikhil Suresh, Umut A. Utku, Justin D. Yao, Todd M. Mowery

**Affiliations:** ^1^Department of Head and Neck Surgery & Communication Sciences, Rutgers Robert Wood Johnson Medical School, New Brunswick, NJ, United States; ^2^Rutgers Brain Health Institute, New Brunswick, NJ, United States

**Keywords:** balance, dizziness, perilymph fistula, sound-induced dizziness, spatial disorientation, superior semicircular canal dehiscence

## Abstract

**Background:**

Superior semicircular canal dehiscence (SSCD) is a vestibular-cochlear disorder in humans in which a pathological third mobile window of the otic capsule creates changes to the flow of sound pressure energy through the perilymph/endolymph. The primary symptoms include sound-induced dizziness/vertigo, inner ear conductive hearing loss, autophony, headaches, and visual problems. We have developed an animal model of this human condition in the Mongolian Gerbil that uses surgically created SSCD to induce the condition. A feature that is unique in this model is that spontaneous resurfacing of the dehiscence occurs via osteoneogenesis without a subsequent intervention. In this study, we completed our assessment of this model to include reversible asymmetrical vestibular impairments that interfere with balance.

**Methods:**

Adult Mongolian gerbils (*N* = 6) were trained to complete a balance beam task. They were also trained to perform a Rotarod task. After 10 days of training, preoperative ABR and c+VEMP testing was followed by a surgical fenestration of the left superior semicircular canal. Balance beam testing recommenced at postoperative day 6 and continued through postoperative day 15 at which point final ABR and c+VEMP testing was carried out.

**Results:**

Behavioral comparison of preoperative and postoperative performance show a significant decrease in Rotarod performance, increased rates of falling, and an increase in time to cross the balance beam. Impairments were the most significant at postoperative day 7 with a return toward preoperative performance by postoperative day 14. This behavioral impairment was correlated with residual impairments to auditory thresholds and vestibular myogenic amplitudes at postoperative day 14.

**Conclusion:**

These results confirm that aberrant asymmetric vestibular output in our model of SSCD results in reversible balance impairments. The level of these behavioral impairments is directly correlated with severity of the vestibular dysfunction as we have previously reported for peripheral ear physiology and cognition.

## Introduction

Superior semicircular canal dehiscence (SSCD) is an inner-ear disorder characterized by a pathological third mobile window in the superior semicircular canal. First described by Minor et al. (1), SSCD presents with a variety of auditory and vestibular symptoms, including autophony, inner ear conductive hearing loss, and sound-induced dizziness/vertigo. These symptoms are often triggered by alterations in middle ear pressure or auditory stimulation (1–4). The precise mechanism by which SSCD develops remains incompletely understood. It is thought to arise from developmental abnormalities or acquired changes in bone density. The diagnosis of SSCD typically involves a combination of clinical history, physical examination, and radiologic imaging, such as high-resolution temporal bone computed tomography and vestibular evoked myogenic potentials (cervical VEMPs [cVEMPs] and ocular VEMPs [oVEMPs]) (5–14).

Previous research has expanded our understanding of the effects of vestibular dysfunction beyond the known limitations in balance, coordination, and spatial orientation. A number of studies have shown that individuals with asymmetric vestibular disorders often exhibit cognitive deficits, including impairments in attention, memory, and executive function, as well as depression (9, 15, 16). This association highlights the intricate relationship between the vestibular system and the central nervous system and warrants further investigation into the governing pathways and potential therapeutic implications of these systems.

The development of animal models has provided valuable insights into the underlying mechanisms of the various signs and symptoms observed in patients. We recently published a gerbil model of SSCD with reversible diagnostic findings characteristic of patients with the disorder (17). Other animal models of SSCD have also been created in chinchillas, guinea pigs, and rats (18–25). These models have contributed to our understanding of the pathophysiology of SSCD and have facilitated the evaluation and diagnosis of the disease. Despite these advancements, the existing models have limitations, including species-specific differences in anatomy and behavior, that warrant the establishment of animal models that more closely mimic the human condition.

The objective of this study was to expand upon our novel gerbil model of SSCD to further encompass the vestibular deficits associated with this condition. Our previous research established the gerbil model of SSCD using auditory brainstem responses (ABR) and cervical positive vestibular evoked myogenic potentials (c+VEMP), with a particular focus on capturing the cognitive impairments commonly seen in humans afflicted with this disorder and other sites of dehiscence resulting in third window syndrome (9, 10, 17, 26, 27). We have previously shown that our SSCD model results in reversible impairments in specific auditory and visual behavioral tasks assessing decision-making, suggesting a potential link between vestibular dysfunction and cognitive deficits (26). Animals with SSCD also show reversible deficits in a spatial two alternative force choice (2AFC) task where they must make a left versus right decision to receive a food reward (28). Furthermore, in that study we used neuroanatomical tracing to confirm a cross species (gerbil and mouse) vestibular behavioral circuit that modulates associative-conditioned tasks through thalamic input to the striatum. Together, these findings show how important proper vestibular function is to normal behaviors. The model provides a powerful approach for studying the central neural etiology of the cognitive behavioral symptoms reported in the human condition (9).

The same surgical techniques used in our previous studies were employed to create the SSCD in a new group of gerbils, with a focus on translating the vestibular deficits, in particular the sound-induced vestibular dysfunction (Tullio phenomenon). In our study, gerbils were trained and tested on the balance beam and Rotarod to assess vestibular function. We observed significant effects of SSCD on time on the Rotarod, time to cross the balance beam, and on falls off the beam. Furthermore, the severity of this balance behavioral impairment was highly correlated with auditory thresholds and vestibular output (c+VEMP amplitudes). By combining physiological measures (ABR and c+VEMP) with direct vestibular testing, we sought to provide a more comprehensive animal model of SSCD that could serve as a foundational model for further study of SSCD and central processes subserving the observed behavioral changes.

## Materials and methods

### Animals

A total of 6 adult male and female Mongolian gerbils *Meriones unguiculatus* (3 males and 3 females) were used in this study. All animals were housed in the same vivarium facility under a 12/12 dark cycle with *ad libitum* access to food and water. All animals were trained to perform the behavioral balance tasks and completed electrophysiologic testing preoperatively. The animals then received superior semicircular canal fenestration to create the SSCD. All experiments were reviewed and approved by Rutgers University Institutional Animal Care and Use Committee.

### Surgical creation of superior semicircular canal dehiscence

Animals were anesthetized with isoflurane and prepared for stereotaxic surgery. An incision was made over the nuchal muscles on the left side of the head just posterior to the pinna. The nuchal muscles were then sharply and bluntly dissected to expose the left superior bulla. A 5.0 mm opening was made with a 1.5 mm diamond bur. For the SSCD surgery, a 2.0 mm fenestration of the labyrinthine bone was made on the apex of the superior canal, without violating the endolymphatic duct. The open bulla was then sealed with Sterile Silastic (Dow Chemical Company, Midland, MI, USA) to partition the air-filled bulla from the overlying neck muscles thereby restoring the normal air-filled middle ear and avoiding a true conductive hearing loss. Condensation on the Silastic seal’s interior surface was deemed indicative of this restoration of function. Finally, the reattached muscles were glued to the skull with Medbond tissue glue (Stoelting Co., Wood Dale, IL, USA) which allowed for c+VEMP testing after the procedure. The incision was closed with a running locked 4–0 Vicryl suture (Ethicon US, LLC, New Brunswick, NJ, USA) and topical antibiotic was applied to the wound.

### Balance beam task

The balance beam apparatus (Panlab Harvard Apparatus, Barcelona, Spain) consisted of a wide beam (6 cm by 120 cm), elevated above the ground, with a covered safe box located at one end. Gerbils were placed on the beam and their ability to maintain balance while traversing its length was observed and recorded using an overhead camera. The SMART 3.0 video tracking software (Panlab Harvard Apparatus, Barcelona, Spain) was employed to precisely measure parameters such as latency to the box and to fall, providing quantitative data for statistical analysis. A multi-field speaker (MF1, Tucker-Davis Technologies, Alachua, FL, USA) was placed 5 cm from the starting position, elevated 6 inches directly above using a tripod. Infrared beams were placed under the speaker to trigger the playback of 90 dB broadband noise from the speaker for 3 s. Gerbils were trained on the balance beam for 3 days and then underwent recorded trials for 5 days before surgery. Testing resumed after surgery at days 6–8 and 13–15 denoted as postoperative 7 and postoperative day 14, respectively, throughout the manuscript and Figures.

### Rotarod task

The Rotarod (Med Associates Inc., Fairfax, VT, USA) was used to further assess vestibular function and balance. The gerbils were placed on the rod, which accelerated from 3 to 30 rpm over 2 min in a linear manner. The length of time on the rod was recorded with a maximum cutoff of 5 min. At 5 min animals were removed from the Rotarod and returned to the cage. Animals were tested three times daily in the morning at least 2 h prior to the balance beam test.

### Auditory brainstem response testing

Animals were anesthetized with isoflurane (1.0%) and placed in a small sound chamber (IAC, Sound Room Solutions, Inc., Glen Cove, NY, USA). Auditory brainstem response (ABR) recordings were made by inserting pin electrodes subcutaneously at the vertex of the skull and just caudal to the right pinna; the ground electrode was inserted into the base of the tail. BioSigRZ software and the TDT ABR system (Tucker-Davis Technologies, Alachua, FL, USA) were used to collect ABR data. A 10-cm tube (closed field) was inserted into the ear and placed at the opening of the ear canal. The left ear of the animal was stimulated via multi-field speaker (MF1, Tucker-Davis Technologies, Alachua, FL, USA) at 1, 2, 4, 8, and 16 kHz tones [90–20 dB SPL (10 dB steps)], 5 ms, 2 ms linear ramp rise-fall times at 25 Hz. Traces were averaged across 512 (threshold) sweeps. Thresholds for each frequency were measured as the last dB SPL (i.e., 10 dB SPL resolution stimulus level) that elicited a tone-induced ABR.

### Sound-induced cervical positive vestibular evoked myogenic potential (c+VEMP) testing

Sound-induced otolithic stimulation and evoked intramuscular excitatory potential recordings were collected by inserting pin electrodes into the neck extensor muscles (splenius capitus *m.*), with the reference electrode placed at the vertex of the skull. BioSigRZ software and the TDT ABR system were used to collect c+VEMP data. A 10-cm tube capable of delivering 100 dB SPL (see TDT specs, Closed Field) was inserted into the ear and placed at the opening of the ear canal. The left ear of the animal was stimulated via multi-field speaker (MF1, Tucker-Davis Technologies) at 2 kHz (100 to 80 dB SPL [5 dB steps], 5 ms, 2 ms linear ramp rise-fall times sampled at 25 kHz). Traces were averaged across 512 (threshold) sweeps. The c+VEMPs were recorded under low-isoflurane anesthesia (<1.5%), near conditions of wakefulness. The c+VEMP was measured when it appeared under the condition of stimulation of air-conducted sound at 2 kHz and 100 dB. Peak amplitudes were measured by subtracting the peak of the negative N1 wave (in μV) from the later positive P1 wave.

### Condenser brightfield stereomicroscopy

After the final ABR and c+VEMP recordings were collected at postoperative day 14, the animals were euthanized (Euthasol 300 mg/kg) and perfused for histology. Each animal’s heart was accessed through the diaphragm. The right atrium was cut, and 20 mL of room-temperature phosphate-buffered saline (1 M) was perfused through the left ventricle. This was followed by 20 mL of cold paraformaldehyde (4%). After perfusion, the animals were decapitated. The left bulla was dissected and immersed in paraformaldehyde (4%). The superior (anterior) semicircular canal was imaged using a condenser brightfield stereomicroscope through the opening into the bulla on a Revolve R4 microscope (ECHO, San Diego, CA, USA). A scale bar was added to each image in the ECHO annotation software. Images were exported to Canvas X for analysis of the dehiscence site. The percentage of bone regrowth via osteoneogenesis was derived as a ratio of the original 2.0 mm fenestration. For each animal the bone regrowth was classified between 0 and 100% as the percentage of 2.0 mm regrowth as follows: 0% (no regrowth), 1–25% (0.1–0.49 mm), 26–49% (0.50–0.99 mm), 50–74% (1.0–1.49 mm), 75–99% (1.5–1.99 mm), full resurfacing was 100% (2.0 mm). Given the interval of time (postoperative day 14) all animals fell into easily discernable categories between 25 and 75% regrowth/resurfacing.

### Statistical analysis

Statistical analyses were performed using JMP software (SAS, Carey, NC, USA) and SPSS (IBM, Armonk, New York, USA). Figures were generated using JMP software. To test the main effects of SSCD on balance beam and Rotarod performance ANOVAs with *post-hoc* analysis using the Tukey Honestly Significant Difference (HSD) test was used. A linear regression analysis was used to calculate adjusted *R*^2^ scores when correlating data within groups. For all analyses, statistical significance was determined at the *p* < 0.05 level or greater. Data in the Figures display group mean ± SEM or actual data points (e.g., regression analysis).

## Results

In this study we used 3 male and 3 female Mongolian gerbils (*Meriones unguiculatus*) that had received a semicircular canal dehiscence to explore vestibular paradigm balance impairments. None of these animals demonstrated circling behavior or complete loss of hearing after creation of the SSCD. Animals were pretrained on a Rotarod and a wide balance beam task, followed by SSCD. Retesting occurred on postoperative day 5–7 (Post_7_) and 13–15 (Post_14_), which represent the peak dysfunction and beginning of recovery from physiological impairment, respectively.

### Superior semicircular canal dehiscence-induced balance impairments during balance beam and Rotarod tasks

**Figure 1:**
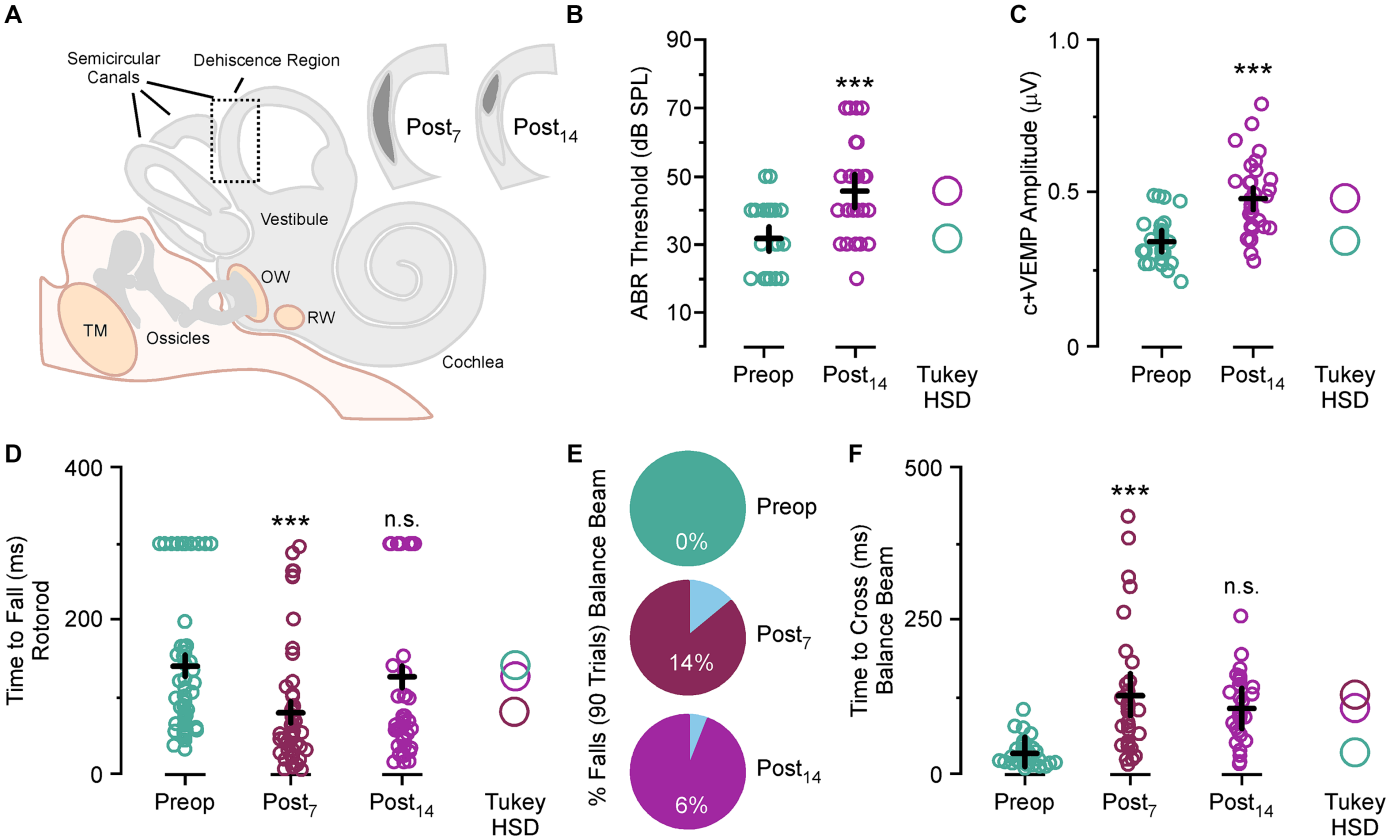
Superior semicircular canal dehiscence (SSCD)-induced balance impairment. **(A)** Diagram showing the location, size, and spontaneous bone regrowth/resurfacing of the dehiscence at postoperative day 7 and 14. **(B)** Scattergram showing a significant increase in ABR thresholds at postoperative day 14. **(C)** Scattergram showing a significant increase in c+VEMP amplitudes at postoperative day 14. **(D)** Scattergram showing significantly faster fall times for the Rotarod task at postoperative day 7 and slight impairment at postoperative day 14 compared to preoperative fall times. **(E)** Pie charts showing the rate of balance beam falls at postoperative day 7 and 14 compared to preoperative rates. **(F)** Scattergram showing the significantly longer times to cross on the balance beam task at postoperative days 7 and 14 compared to preoperative crossing times. ABR, auditory brainstem response; c+VEMP, cervical positive vestibular evoked potential; dB SPL, decibel sound pressure level; n.s., not significant; ms, millisecond; OW, oval window; Post_7_, postoperative day 7 (dark purple); Post_14_, postoperative day 14 (light purple); Preop, preoperative (teal); R^2^_adjust_, adjusted r-squared; RW, round window; TM, tympanic membrane; Tukey HSD, Tukey Honestly Significant Difference. ***p < 0.001.

Animals were trained to cross a balance beam without falling off or tested on time to fall on a Rotarod task (Figure 1). After five testing sessions the experimental animals had a 2.0 mm semicircular canal dehiscence created (Figure 1A). This was used to induce an asymmetric vestibular impairment, which was still present at postoperative day 14 as an inner ear conductive hearing loss that produces elevated auditory thresholds across all frequencies (Mean ± SEM; preoperative thresholds 31.6 ± 2.1 vs. postoperative day 14 thresholds 45.6 ± 2.5, *p* < 0.001) (Figure 1B) and increased c+VEMP amplitudes at 2 kHz stimulation (preoperative amplitudes 340 ± 17 μv vs. postoperative day 14 amplitudes 480 ± 24 μv, *p* < 0.001) (Figure 1C).

For each day of testing, the animal was placed on the far end of the beam and completed the task by running the beam’s length to the enclosure on the opposite end. This was repeated and measured five times. There was a significant increase in time to cross with each trial compared to the first trial for preoperative testing, postoperative testing at day 7 and day 14 (Mean ± SEM; Trial 1, 50.9 ± 7.2 ms vs. Trial 2, 60.9 ± 7.7 ms vs. Trial 3, 86.6 ± 10.0 ms vs. Trial 4, 111.2 ± 13.1 ms vs. Trial 5, 138.6 ± 16.4 ms, *p* values all <0.05). This amounted to a group average increase in time to cross for Trial 2 at 64%, Trial 3 at 158%, Trial 4 at 221% and trial 5 at 368%. This trend was seen in every animal before and after surgical creation of the SSCD.

For this task a noise stimulus was played after the animal broke a beam five centimeters from the starting platform. In our previous study we show a significant increase in sound driven (2 kHz tone) c+VEMP amplitude (17) that is indicative of stimulus evoked asymmetrical vestibular output. This occurs through shunting of the sound wave energy via the dehiscence, which also dampens cochlear hair cell activation and leads to the inner ear conductive hearing loss. We hypothesized that this phenomenon might contribute to symptom expression, so we designed the experiment to assess whether noise-induced changes could be detected behaviorally in the SSCD animal model. In preoperative training the noise stimulus was introduced to reduce startle effects to the noise after SSCD. For each five-trial session the stimulus was triggered on Trial 3. As we saw a linear function in the increase in time to cross for each travel, we did not see a significant effect of noise on preoperative travel times (Trial 2, 23.6 ± 3.7 ms vs. Trial 3, 27.7 ± 3.9 ms; *p* > 0.1; Trial 3, 27.7 ± 3.9 ms vs. Trial 4, 37.8 ± 5.0 ms; *p* > 0.1). For postoperative trials the noise stimulus was played on Trial 1, 2, 4 and 5. Thus to test for a significant effect of noise onset after SSCD we would expect to see faster travel times for Trial 3 compared to Trial 2; however, we only observed the linear increase from Trial 2 to Trial 3 for postoperative day 7 (Trial 2, 89.4 ± 16.4 ms vs. Trial 3, 121.4 ± 20.8 ms; *p* > 0.1) and postoperative day 14 (Trial 2, 69.7 ± 14.2 ms vs. Trial 3, 110.7 ± 16.1 ms; *p* > 0.1) seen in preoperative testing. This suggested the noise stimulus induced no delay on time to cross and the non-noise trial did not show any savings in time to cross.

Despite not having a noise effect, we did observe impaired balance postoperatively (Figure 1D). For the Rotarod task, animals fell more often (preoperative fall rate, 79% vs. postoperative day 7 fall rate, 96%) and significantly faster (Mean ± SEM preoperative time to fall, 141.4 ± 12.6 ms vs. postoperative day 7, 80.6 ± 12.1, *p* < 0.001) at postoperative day 7. There was no significant difference in fall rate (preoperative fall rate, 79% vs. postoperative day 14 fall rate, 70%) or time to fall (Mean ± SEM preoperative time to fall, 141.4 ± 12.6 ms vs. postoperative day 14, 126.7 ± 16.0, *p* > 0.1) at postoperative day 14. The bimodal distribution of the postoperative day 14 data is indicative of differences in the rate of bone resurfacing between animals as we have previously noted (17). Removal of outlier sessions where the animals stayed on the Rotarod throughout the entire session shows that compared to preoperative time to fall there were significant increases in fall time for animals that did fall at postoperative day 14 (Mean ± SEM preoperative time to fall, 96.1 ± 6.5 ms vs. postoperative day 14, 54.8 ± 6.4, *p* < 0.01).

For the wide beam balance task, not a single animal fell preoperatively over 90 trials; however, we did see fall rates of 14 and 6% after SSCD. These falls always happened on noise stimulus trials; however, incidence rates were too low to draw any conclusions about sound-induced falling. In Figure 1E, we show that there was a significant adverse effect of SSCD on time to cross at postoperative day 7 (Mean ± SEM; preoperative time to cross 34.2 ± 22 ms vs. postoperative day 7 time to cross 128.3 ± 34 ms, *p* < 0.001) and postoperative day 14 (preoperative time to cross 34.2 ± 22 ms vs. postoperative day 14 time to cross 106.3 ± 28 ms, *p* < 0.001). It did take the animals longer to cross on postoperative day 7 vs. postoperative day 14, but this was not significant (postoperative day 7 time to cross 128.3 ± 34 ms, *p* < vs. postoperative day 14 time to cross 106.3 ± 28 ms, *p* > 0.1). We found the same trend with the Rotarod studies (Figure 1F). Again, the time to fall for postoperative day 7 was significantly faster than preoperative times (preoperative time to fall 141 ± 13 ms vs. postoperative day 7 time to fall 80 ± 34 ms, *p* < 0.001). There was a faster fall time at postoperative day 14; however, this was not significant (preoperative time to fall 141 ± 13 ms vs. postoperative day 14 time to fall 126 ± 27 ms, *p* > 0.1). Time to fall was significantly faster at postoperative day 7 compared to postoperative day 14 (postoperative day 7 time to fall 80 ± 34 ms vs. postoperative day 14 time to fall 126 ± 27 ms, *p* < 0.05). All together, we found an effect of SSCD on balance; and the impact on balancing behavior is highest at the peak of the SSCD induced physiological, spatial, and cognitive impairments we have previously reported (17, 26, 28).

### ABR and c+VEMP measurements correlate with balance impairment

**Figure 2:**
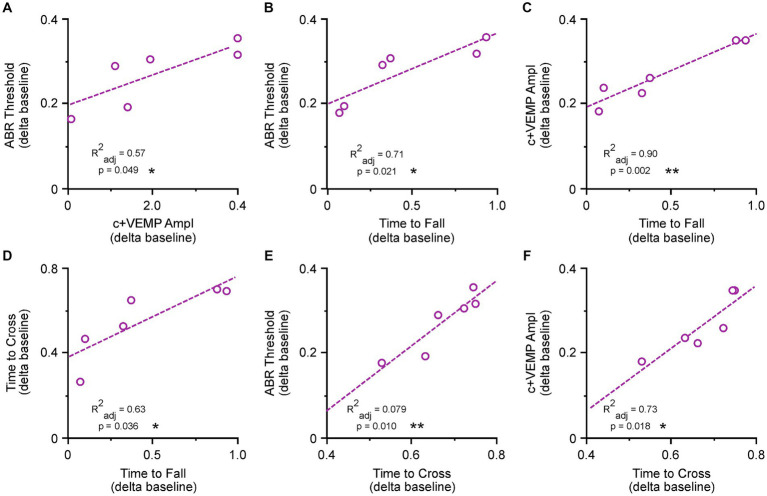
Correlations between superior semicircular canal dehiscence (SSCD)-induced changes to peripheral inner ear function and behavioral balance performance at postoperative day 14 (light purple). **(A)** Scatterplot showing correlation between ABR thresholds and c+VEMP amplitudes. **(B)** Scatterplot showing correlation between ABR thresholds and time to fall in the Rotarod task. **(C)** Scatterplot showing correlation between c+VEMP amplitudes and time to fall in the Rotarod task. **(D)** Scatterplot showing correlation between time to cross in the balance beam task and time to fall in the Rotarod task. **(E)** Scatterplot showing correlation between ABR thresholds and time to cross in the balance beam task. **(F)** Scatterplot showing correlation between c+VEMP amplitudes and time to cross in the balance beam task. ABR, auditory brainstem response; c+VEMP, cervical positive vestibular evoked potential; R^2^_adjust_, adjusted r-squared. *p < 0.05, **p < 0.01.

To verify the involvement of vestibular dysfunction with balance beam and Rotarod measured impairments we correlated the ABR and c+VEMP thresholds and amplitudes as a function of physiological severity versus behavioral impairment at the end of postoperative testing (Figure 2). ABR and c+VEMP measurements were not recorded for postoperative day 6–8 as the animals are very sensitive to anesthesia and we did not want these affects to influence behavior. We also did not include c+VEMP threshold data as these occur below the lowest dB SPL measured by our apparatus (20 dB SPL) in our animal model. As previously reported (17) we observed a significant correlation between each animal’s auditory threshold and their c+VEMP amplitude (Figure 2A). These are measured as percentage postoperative changes from preoperative baselines (ABR = 0.4 + 0.84 × c+VEMP; adjusted r-squared = 0.57, *p* < 0.05). There is also a correlation between ABR thresholds and time to fall during Rotarod testing at postoperative day 14 (ABR = 0.19–0.16 × time to fall; adjusted r-squared = 0.71, *p* < 0.05) (Figure 2B). There was also a correlation between ABR thresholds and time to fall during Rotarod testing at postoperative day 14 (ABR = 0.19–0.16 × time to fall; adjusted r-squared = 0.71, *p* < 0.05) (Figure 2B). A much stronger correlation is seen for c+VEMP amplitudes and time to fall from the Rotarod at postoperative day 14 (c+VEMP = 0.18–0.17 × time to fall; adjusted r-squared = 0.90, *p* < 0.01) (Figure 2C). The time to fall from the Rotarod was correlated with the time to cross the balance beam for postoperative day 14 animals (time to cross = 0.58–0.18 × time to fall; adjusted r-squared = 0.63, *p* < 0.05) (Figure 2D). The same trends that were observed for time to fall on the Rotarod apply to time to cross on the wide balance beam (Figures 2E,F). There was a significant correlation between ABR thresholds and time to cross at postoperative day 14 (ABR threshold = 0.25 + 0.78 × time to cross; adjusted r-squared = 0.79, *p* < 0.05) as well as between c+VEMP and time to cross (c+VEMP = −0.22 + 0.72 × time to cross; adjusted r-squared = 0.73, *p* < 0.05). All together these correlations showed a strong connection between SSCD-induced changes to peripheral physiology and Rotarod and balance beam performance impairment.

### Condenser brightfield stereomicroscopy

**Figure 3:**
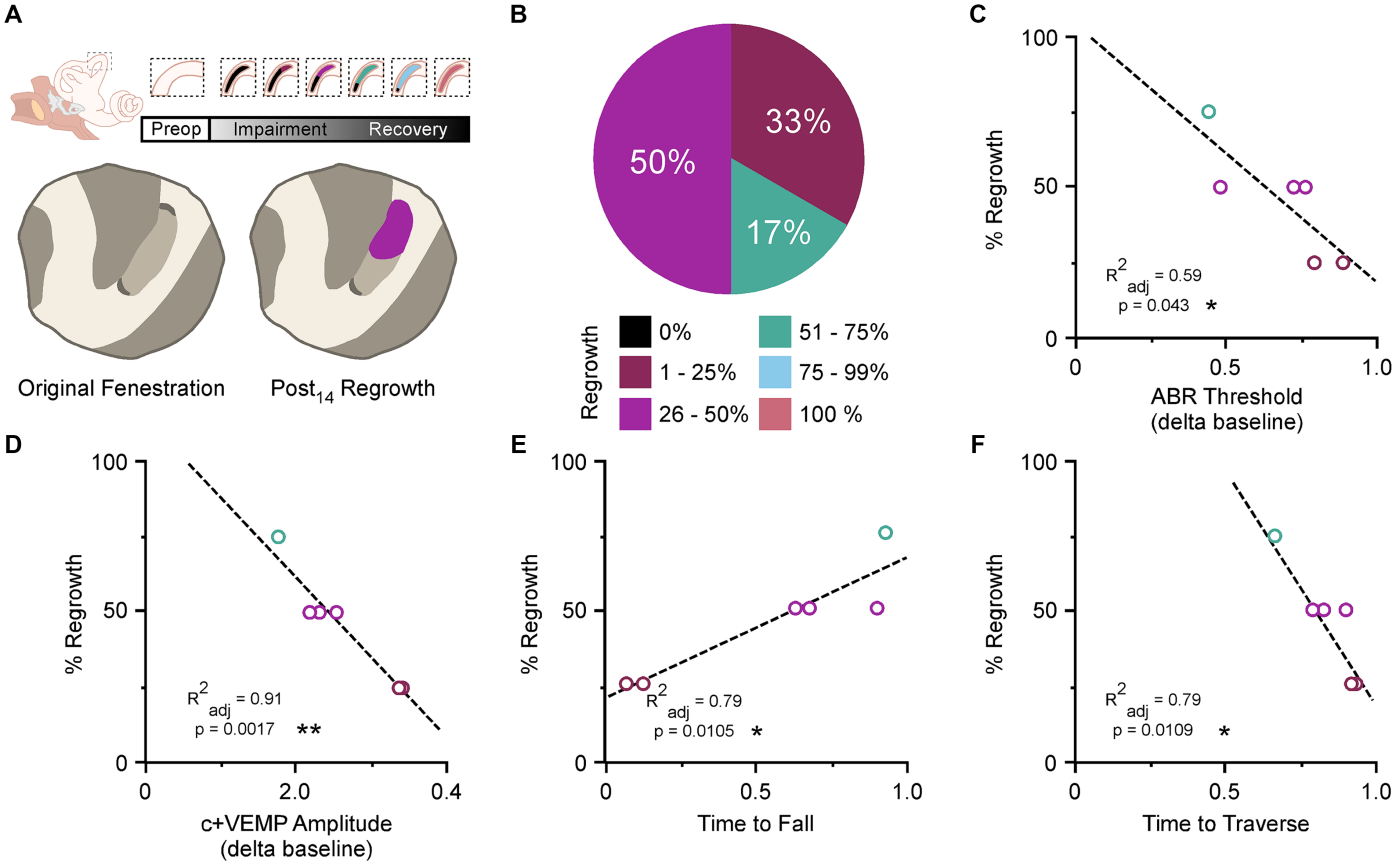
Correlation between bone regrowth, behavioral, and physiological impairment. **(A)** Diagram showing the general association between bone resurfacing and physiological impairment (*top*). Cartoon showing example of original fenestration and the typical regrowth associated with postoperative day 14 (*bottom*). **(B)** Pie chart showing the percentage of regrowth in fenestrated canals at each level of classification for postoperative day 14. **(C)** Correlation between percentage of bone regrowth and the percent ABR threshold shifts at postoperative day 14. **(D)** Correlation between percentage of bone regrowth and the percent c+VEMP amplitude shifts at postoperative day 14. **(E)** Correlation between percentage of bone regrowth and the time to fall on the Rotarod task at postoperative day 14. **(F)** Correlation between percentage of bone regrowth and the time to cross the balance beam at postoperative day 14. %, percentage; ABR, auditory brainstem response; c+VEMP, cervical positive vestibular evoked potential; Post_14_, postoperative day 14; Preop, preoperative. *p < 0.05, **p < 0.01.

To verify the involvement of bone regrowth status with physiological and behavioral impairments at postoperative day 14 we correlated ABR thresholds, c+VEMP amplitudes, time to fall on the Rotarod task, and time to traverse the beam task (Figure 3). As previously reported (17), bone regrowth/resurfacing via osteoneogenesis commences shortly after fenestration with the bone being completely resurfaced by 1 month (Figure 3A). The status of bone regrowth at postoperative day 14 is typically between 25 and 50%. In the current study we confirmed the same trend (Figure 3B). Again, we saw significant correlations between the percentage of bone regrowth and ABR thresholds (regrowth = 1.04–2.15 × ABR threshold; adjusted r-squared = 0.59, *p* < 0.05) (Figure 3C) and a highly significant correlation for c+VEMP amplitudes (regrowth = 1.15–2.63 × c+VEMP amplitude; adjusted r-squared = 0.91, *p* < 0.01) (Figure 3D). For the Rotarod task there was a significant correlation between percentage bone regrowth and time to fall (regrowth = 0.66–0.45 × time to fall; adjusted r-squared = 0.79, *p* < 0.05) (Figure 3E). Finally, for the balance beam task there was a significant correlation between percentage of bone regrowth and time to traverse (regrowth = 1.83–2.03 × time to traverse; adjusted r-squared = 0.79, *p* < 0.05) (Figure 3F).

## Discussion

In patients with SSCD, sound-induced symptoms are a common component of their clinical presentation (13, 14, 29). Often referred to as the Tullio phenomenon, sound-induced vestibular dysfunction is the result of abnormal shunting of sound pressure from the cochlea out through the dehiscent superior semicircular canal, creating a pathologic third mobile window (9, 28–33). Studies have demonstrated that in addition to inner ear conductive hearing loss, patients exhibit these balance impairments and vertigo from sudden, loud sounds along with abnormal vestibular-evoked myogenic potentials (VEMPs) (5, 6, 9, 13, 18, 34–37). Surgical plugging and correction of the dehiscence often leads to the resolution of sound-induced symptoms with preserved hearing thresholds (5, 9, 38–40).

In addition to our previous work developing the gerbil SSCD model (17, 26), other animal models have effectively demonstrated and explored the mechanisms behind the auditory deficits, namely the inner ear conductive hearing loss found in SSCD, in fat sand rats, guinea pigs and chinchillas (18–22, 41). Studies in mice have also investigated the effect of different vestibular lesions (labyrinthectomy, absent otoliths) on Rotarod and balance beam performance (42–47). To our knowledge, the present study is the first to directly assess and reproduce the vestibular deficits found in SSCD.

In this report we expanded upon the auditory and vestibular electrophysiology findings as well as the cognitive and spatial behavioral impairments observed in our animal model of SSCD (17, 26, 28). With our model established to date, the only remaining elements needed to replicate the human experience with SSCD was to demonstrate vestibular dysfunction and sound-induced vestibular dysfunction.

After training the animals to balance on a Rotarod and to cross a wide beam, we completed the surgical creation of a SSCD and subsequently tested balance behavioral impairments during the peak (postoperative day 7) and beginning of recovery from the pathological third mobile window (postoperative day 14). We found significant effects of SSCD on time on the Rotarod, time to cross the balance beam, and on falls off the beam. Finally, the severity of this balance behavioral impairment was highly correlated with auditory thresholds and otolithic vestibular evoked responses (c+VEMP amplitudes). Together, these findings suggests that the recovery status of the dehiscence (and how large it is) will determine the severity of the balance impairments in this model. This makes it an ideal model for testing a wide variety of hypotheses associated with asymmetric vestibular dysfunction in humans due to SSCD.

To measure the degree of spontaneous resurfacing of the SSCD, we performed condenser brightfield stereomicroscopy. We then correlated the change on ABR thresholds, change in c+VEMP amplitudes, time to fall during the Rotarod task and the time to traverse during the balance beam task. For all four of these correlations, there was a statistically significant correlation between the degree of resurfacing and the electrophysiological measures and the behavioral measures.

A potential weakness of the present study is that some of the observed behavioral balance impairments could have resulted from postoperative serous labyrinthitis rather than the SSCD alone. Our inclusion–exclusion criteria excluded animals who exhibited persistent circling behavior, poor feeding and drinking behavior and loss of ABR and c+VEMP function. It is also the case that the elevated c+VEMP amplitudes would not be expected if a serous labyrinthitis impaired otolithic function after SSCD. We also minimized this effect by delaying the initial post SSCD testing for 6 days to allow for recovery after the surgical procedure. Administration of corticosteroids postoperatively was considered but not included in the study protocol because effective intralabyrinthine corticosteroid levels in gerbils has not been studied after intraperitoneal delivery, nor has a dosing regimen been studied in these animals.

Another potential weakness of our model is that the temporal lobe dura and temporal lobe is not resting on the SSCD as it likely does in patients with this disorder. An animal model of a human condition is our best approximation of human pathology, which is imperfect. In our model, there is no covering of the surgically created third mobile window, but rather the perilymph is exposed to the humid environment of the reconstructed bulla. In designing our model, we considered covering the dehiscence site; however, we were concerned about inducing an inflammatory reaction and serous labyrinthitis, so we did not do this. We do know from our initial two papers establishing this animal model of SSCD that there are measured changes in ABR and c+VEMP that are reversible much like surgical management of SSCD patients experience, as well as reversible cognitive function changes (17, 26). For these reasons we did not cover the dehiscence site in the present series of experiments.

In our gerbil model of SSCD, the experimental reproduction of sound-induced vestibular symptoms would have served as a more accurate representation of human symptoms. While we observed more sound-induced dysfunction, these differences did not reach statistical significance. There are two possibilities to explain this, which are likely related. Either the sample size was too small to capture a small effect, or the acute sound exposure was not adequate to elicit the expected sound-induced vestibular dysfunction. One limitation of this study is that the speaker utilized could not consistently produce sound levels above 90 dB or adequately reproduce lower frequencies sound at that dB SPL. Thus, it is possible that an essential frequency range known to induce vestibular dysfunction symptoms in patients with SSCD were not delivered to the gerbils with SSCD (2, 25, 48). Future studies should address this by employing more powerful and lower-frequency sound sources to more accurately model the sound-induced vestibular dysfunction found in patients with SSCD. Future work in this animal model will focus on directly investigating the Tullio phenomenon (nystagmus), as well as the effect of SSCD on cochlear function. The development of scleral search coils for use in gerbils would also provide the opportunity to measure sound-induced nystagmus in this experimental model. Here, we plan to utilize electrocochleography around the timelines established in our vestibular paradigms to investigate how dehiscence will affect cochlear summating potentials and action potentials throughout recovery (resurfacing).

## Conclusion

The findings reported herein help to further establish the Mongolian gerbil as an appropriate model for understanding the specific effects of SSCD on vestibular function. Our gerbil model reproduced the vestibular deficits seen in patients with SSCD, as evidenced by increased beam-crossing times and increased Rotarod times on postoperative day 7 (acute phase of impairment) with improvement corresponding to the onset of dehiscence recovery. These findings build upon the gerbil SSCD model showing an increase in both ABR thresholds, as a proxy for inner ear conductive hearing loss, and elevated c+VEMP amplitudes, mimicking the abnormal physiological measures found in the human disorder along this same timeline. We subsequently demonstrated that these measures, specifically c+VEMP amplitudes, had negative and reversible correlations with decision-making, suggesting potential CNS deficits resulting from the SSCD, again along this 7-day peak with the onset of recovery corresponding to behavior returning toward preoperative levels. Finally, even though we do not observe increased sound-induced vestibular dysfunction, it should be noted that a general impairment to balance is present regardless of auditory stimulation, and this is also what patients with SSCD experience. This finding is similar to the cognitive and spatial behavioral impairments which is also what patients with SSCD experience, which do not require sound-induced stimulus to become manifest.

## Data Availability

The raw data supporting the conclusions of this article will be made available by the authors, without undue reservation.

## References

[1] Minor LB Solomon D Zinreich JS Zee DS. Sound- and/or pressure-induced vertigo due to bone dehiscence of the superior semicircular canal. Arch Otolaryngol Head Neck Surg. (1998) 124:249–58. doi: 10.1001/archotol.124.3.249, PMID: 9525507

[2] Cremer PD Minor LB Carey JP Della Santina CC. Eye movements in patients with superior canal dehiscence syndrome align with the abnormal canal. Neurology. (2000) 55:1833–41. doi: 10.1212/WNL.55.12.1833, PMID: 11134382

[3] Minor LB. Clinical manifestations of superior semicircular canal dehiscence. Laryngoscope. (2005) 115:1717–27. doi: 10.1097/01.mlg.0000178324.55729.b7, PMID: 16222184

[4] Merchant SN Rosowski JJ. Conductive hearing loss caused by third-window lesions of the inner ear. Otol Neurotol. (2008) 29:282–9. doi: 10.1097/MAO.0b013e318161ab24, PMID: 18223508 PMC2577191

[5] Welgampola MS. Evoked potential testing in neuro-otology. Curr Opin Neurol. (2008) 21:29–35. doi: 10.1097/WCO.0b013e3282f39184, PMID: 18180649

[6] Watson SR Halmagyi GM Colebatch JG. Vestibular hypersensitivity to sound (Tullio phenomenon): structural and functional assessment. Neurology. (2000) 54:722–8. doi: 10.1212/WNL.54.3.722, PMID: 10680810

[7] Belden CJ Weg N Minor LB Zinreich SJ. CT evaluation of bone dehiscence of the superior semicircular canal as a cause of sound- and/or pressure-induced vertigo. Radiology. (2003) 226:337–43. doi: 10.1148/radiol.2262010897, PMID: 12563123

[8] Wackym PA Wood SJ Siker DA Carter DM. Otic capsule dehiscence syndrome: superior canal dehiscence syndrome with no radiographically visible dehiscence. Ear Nose Throat J. (2015) 94:E8–9. doi: 10.1177/014556131509400802, PMID: 26322461

[9] Wackym PA Balaban CD Mackay HT Wood S Lundell C Carter D. Longitudinal cognitive and neurobehavioral functional outcomes before and after repairing otic capsule dehiscence. Otol Neurotol. (2016) 37:70–82. doi: 10.1097/MAO.0000000000000928, PMID: 26649608 PMC4674143

[10] Wackym PA Balaban CD Zhang P Siker DA Hundal JS. Third window syndrome: surgical management of cochlea-facial nerve dehiscence. Front Neurol. (2019) 10:1281. doi: 10.3389/fneur.2019.01281, PMID: 31920911 PMC6923767

[11] Wackym PA Balaban CD Ikezono T Agrawal Y, (Eds). (2021). Third window syndrome. Lausanne: Frontiers Media SA, pp. 1–230. 10.3389/fneur.2021.704095 PMC8250852 34220698

[12] Schwartz T Lindemann TL Mongelluzzo G Wackym PA Gadre AK. Gray-scale inversion on high resolution computed tomography of the temporal bone: an observational study. Ann Otol Rhinol Laryngol. (2021) 130:1125–31. doi: 10.1177/0003489421996844, PMID: 33629604

[13] Ward BK Carey JP Minor LB. Superior canal dehiscence syndrome: lessons from the first 20 years. Front Neurol. (2017) 8:177. doi: 10.3389/fneur.2017.00177, PMID: 28503164 PMC5408023

[14] Naert L van R van P Bisdorff A Sharon J Ward B. Aggregating the symptoms of superior semicircular canal dehiscence syndrome. Laryngoscope. (2018) 128:1932–8. doi: 10.1002/lary.27062, PMID: 29280497

[15] Smith PF Zheng Y. From ear to uncertainty: vestibular contributions to cognitive function. Front Integr Neurosci. (2013) 7:84. doi: 10.3389/fnint.2013.00084, PMID: 24324413 PMC3840327

[16] Popp P Wulff M Finke K Rühl M Brandt T Dieterich M. Cognitive deficits in patients with a chronic vestibular failure. J Neurol. (2017) 264:554–63. doi: 10.1007/s00415-016-8386-7, PMID: 28074268

[17] Wackym PA Balaban CD Van Osch OJ Morris B Tamakloe MA Salvatore V. New model of superior semicircular canal dehiscence with reversible diagnostic findings characteristic of patients with the disorder. Front Neurol. (2023) 13:1035478. doi: 10.3389/fneur.2022.1035478.;, PMID: 36742050 PMC9892720

[18] Hirvonen TP Carey JP Liang CJ Minor LB. Superior canal dehiscence: mechanisms of pressure sensitivity in a chinchilla model. Arch Otolaryngol Head Neck Surg. (2001) 127:1331–6. doi: 10.1001/archotol.127.11.1331, PMID: 11701069

[19] Carey JP Hirvonen TP Hullar TE Minor LB. Acoustic responses of vestibular afferents in a model of superior canal dehiscence. Otol Neurotol. (2004) 25:345–52. doi: 10.1097/00129492-200405000-00024, PMID: 15129116

[20] Songer JE Rosowski JJ. A mechano-acoustic model of the effect of superior canal dehiscence on hearing in chinchilla. J Acoust Soc Am. (2007) 122:943–51. doi: 10.1121/1.2747158, PMID: 17672643 PMC2254311

[21] Songer JE Rosowski JJ. A superior semicircular canal dehiscence-induced air-bone gap in chinchilla. Hear Res. (2010) 269:70–80. doi: 10.1016/j.heares.2010.07.002, PMID: 20638462 PMC2936693

[22] Attias J Nageris BI Shemesh R Shvero J Preis M. Superior canal dehiscence effect on hearing thresholds: animal model. Otolaryngol Head Neck Surg. (2011) 145:648–53. doi: 10.1177/0194599811410535, PMID: 21602535

[23] Tong BS He ZY Ding CR Yang JM Wang J Han Z. Mechanisms of hearing loss in a Guinea pig model of superior semicircular canal dehiscence. Neural Plast. (2018) 2018:1258341. doi: 10.1155/2018/1258341, PMID: 29853836 PMC5941760

[24] Dlugaiczyk J Burgess AM Goonetilleke SC Sokolic L Curthoys IS. Superior canal dehiscence syndrome: relating clinical findings with vestibular neural responses from a Guinea pig model. Otol Neurotol. (2019) 40:e406–14. doi: 10.1097/MAO.0000000000001940, PMID: 30870375

[25] Curthoys IS Smith CM Burgess AM Dlugaiczyk J. A review of neural data and modelling to explain how a semicircular canal dehiscence (SCD) causes enhanced VEMPs, skull vibration induced nystagmus (SVIN), and the Tullio phenomenon. Audiol Res. (2023) 13:418–30. doi: 10.3390/audiolres13030037, PMID: 37366683 PMC10294846

[26] Mowery TM Wackym PA Nacipucha J Dangcil E Stadler R Tucker A. Superior semicircular canal dehiscence and subsequent closure induces reversible impaired decision-making. Front Neurol. (2023) 14:1259030. doi: 10.3389/fneur.2023.1259030, PMID: 37905188 PMC10613502

[27] Chari DA Juliano AF Jung DH. Radiologically-proven new development of superior semicircular canal dehiscence associated with development of superior semicircular canal dehiscence syndrome. Otol Neurotol. (2021) 42:285–9. doi: 10.1097/MAO.0000000000002912, PMID: 33273305

[28] Smith JB Hong SS Murphy DJ Dangcil E Nacipucha J Tucker A. Neuroanatomical mapping of the gerbil corticostriatal sensory, motor and thalamostriatal parafascicular nucleus inputs reveals a thalamic relay for vestibular information across the striatum. eNeuro. (2024) 11:ENEURO.0273-24.2024. doi: 10.1523/ENEURO.0273-24.2024, PMID: 39142821 PMC11373881

[29] Baloh RW. Superior semicircular canal dehiscence syndrome: leaks and squeaks can make you dizzy. Neurology. (2004) 62:684–5. doi: 10.1212/01.WNL.0000118644.59800.6A, PMID: 15007114

[30] Basura GJ Cronin SJ Heidenreich KD. Tullio phenomenon in superior semicircular canal dehiscence syndrome. Neurology. (2014) 82:1010. doi: 10.1212/WNL.0000000000000217, PMID: 24638216

[31] Dumas G Curthoys IS Castellucci A Dumas L Perrin P Schmerber S. A bone-conducted Tullio phenomenon – a bridge to understand skull vibration induced nystagmus in superior canal dehiscence. Front Neurol. (2023) 14:1183040. doi: 10.3389/fneur.2023.1183040 37360355 PMC10288865

[32] Ostrowski VB Byskosh A Hain TC. Tullio phenomenon with dehiscence of the superior semicircular canal. Otol Neurotol. (2001) 22:61–5. doi: 10.1097/00129492-200101000-00012, PMID: 11314718

[33] Pullicino R Grech R. Tullio phenomenon in superior semicircular canal dehiscence (SSCD). BMJ Case Rep. (2015) 2015:bcr2015213674. doi: 10.1136/bcr-2015-213674 PMC4691913 26698215

[34] Aw ST Todd MJ Halmagyi GM. Latency and initiation of the human vestibuloocular reflex to pulsed galvanic stimulation. J Neurophysiol. (2006) 96:925–30. doi: 10.1152/jn.01250.2005, PMID: 16641374

[35] Lempert T von Brevern M. Episodic vertigo. Curr Opin Neurol. (2005) 18:5–9. doi: 10.1097/00019052-200502000-00003, PMID: 15655395

[36] Rosengren SM Aw ST Halmagyi GM Todd NP Colebatch JG. Ocular vestibular evoked myogenic potentials in superior canal dehiscence. J Neurol Neurosurg Psychiatry. (2008) 79:559–68. doi: 10.1136/jnnp.2007.126730, PMID: 17766428

[37] Niesten ME McKenna MJ Herrmann BS Grolman W Lee DJ. Utility of cVEMPs in bilateral superior canal dehiscence syndrome. Laryngoscope. (2013) 123:226–32. doi: 10.1002/lary.23550, PMID: 22991076

[38] Shaia WT Diaz RC. Evolution in surgical management of superior canal dehiscence syndrome. Curr Opin Otolaryngol Head Neck Surg. (2013) 21:497–502. doi: 10.1097/MOO.0b013e328364b3ff, PMID: 23989599

[39] Mau C Kamal N Badeti S Reddy R Ying YM Jyung RW. Superior semicircular canal dehiscence: Diagnosis and management. J Clin Neurosci. (2018) 48:58–65. doi: 10.1016/j.jocn.2017.11.019, PMID: 29224712

[40] Michailidou E Rüegg PO Karrer T Korda A Weder S Kompis M. Hearing results after transmastoid superior semicircular canal plugging for superior semicircular canal dehiscence: a meta-analysis. Audiol Res. (2023) 13:730–40. doi: 10.3390/audiolres13050065, PMID: 37887846 PMC10604912

[41] Nageris BI Attias J Shemesh R Hadar T Preis M. A third window of the posterior semicircular canal: an animal model. Laryngoscope. (2010) 120:1034–7. doi: 10.1002/lary.20831, PMID: 20422700

[42] Machado ML Kroichvili N Freret T Philoxène B Lelong-Boulouard V Denise P. Spatial and non-spatial performance in mutant mice devoid of otoliths. Neurosci Lett. (2012) 522:57–61. doi: 10.1016/j.neulet.2012.06.016, PMID: 22705908

[43] Tung VW Burton TJ Quail SL Mathews MA Camp AJ. Motor performance is impaired following vestibular stimulation in ageing mice. Front Aging Neurosci. (2016) 8:12. doi: 10.3389/fnagi.2016.00012, PMID: 26869921 PMC4737917

[44] Kim G Nguyen N Kim KS. Postural control in paw distance after labyrinthectomy-induced vestibular imbalance. Med Biol Eng Comput. (2020) 58:3039–47. doi: 10.1007/s11517-020-02276-9, PMID: 33079344

[45] Shaabani M Lotfi Y Karimian SM Rahgozar M Hooshmandi M. Data on galvanic-evoked head movements in healthy and unilaterally labyrinthectomized rats. Data Brief. (2016) 9:338–44. doi: 10.1016/j.dib.2016.08.048, PMID: 27672673 PMC5030318

[46] Negishi-Oshino R Ohgami N He T Ohgami K Li X Kato M. cVEMP correlated with imbalance in a mouse model of vestibular disorder. Environ Health Prev Med. (2019) 24:39. doi: 10.1186/s12199-019-0794-8 31153359 PMC6545207

[47] Modi AD Parekh A Patel ZH. Methods for evaluating gait associated dynamic balance and coordination in rodents. Behav Brain Res. (2024) 456:114695. doi: 10.1016/j.bbr.2023.114695, PMID: 37783346

[48] Luecke VN Buchwieser L Zu Eulenburg P Marquardt T Drexl M. Ocular and cervical vestibular evoked myogenic potentials elicited by air-conducted, low-frequency sound. J Vestib Res. (2020) 30:235–47. doi: 10.3233/VES-200712, PMID: 32925129

[49] Stewart CE Kanicki AC Altschuler RA King WM. Vestibular short-latency evoked potential abolished by low-frequency noise exposure in rats. J Neurophysiol. (2018) 119:662–7. doi: 10.1152/jn.00668.2017, PMID: 29118200 PMC5867388

